# Acute Psychological Stress Disrupts Attentional Bias to Threat-Related Stimuli

**DOI:** 10.1038/s41598-017-14138-w

**Published:** 2017-11-03

**Authors:** Caihong Jiang, Tony W. Buchanan, Zhuxi Yao, Kan Zhang, Jianhui Wu, Liang Zhang

**Affiliations:** 1 Key Laboratory of Behavioral Science, Institute of Psychology, Chinese Academy of Sciences, Beijing, China; 2 Department of Industrial Engineering, Tsinghua University, Beijing, 100084 China; 3 Department of Psychology, Saint Louis University, St. Louis, MO USA; 4 College of Psychology and Sociology, Shenzhen University, No.3688, Nanhai Rd., Shenzhen, Guangdong, China; 5 Department of Psychology, University of Chinese Academy of Science, Beijing, China

**Keywords:** Attention, Attention, Stress and resilience, Stress and resilience, Human behaviour

## Abstract

The present study investigated the effect of acute stress on attentional bias to threat using behavioral and ERP methods. Sixty-two male participants were randomly assigned to a stress condition (Trier Social Stress Test) or a control condition. To examine the impact of stress-induced cortisol on attentional bias to threat, participants in the stress group were split into Low- and High cortisol responders. All participants were then administered a modified dot probe task in which the cues were neutral and angry faces. Behavioral results showed a pattern of attentional bias toward threat in the Control group but not in the stress group. For the ERPs, the P100 peaked earlier for the angry-cued targets than the neutral-cued targets in the Control group, which suggests a rapid, adaptive response toward threat. However, this effect was not observed in the stress group, suggesting a suppressed attentional bias under stress. In addition, the stress group (including both Low and High cortisol responders) showed reduced P300 amplitude to target onset than the Control group. These results suggest that acute stress disrupts attentional bias to threat including a reduction in early bias to threat in addition to a subsequent change of attention allocation.

## Introduction

People can detect and respond rapidly to threats in their environment. In contrast to a neutral stimulus, this hypervigilance to a threatening stimulus is called attentional bias toward threats^1^. However, this enhanced attention to threat can be altered by stress. Previous studies have found an attentional bias toward threat stimuli in non-stressful contexts. However, the bias may disappear under acute stress. For example, the attentional bias toward threat disappeared when socially anxious individuals were asked to perform public speaking^2^. Likewise, the attentional bias to masked angry face for patients with psychogenic non-epileptic seizures was abolished when they experienced the Trier Social Stress Test (TSST)^3^. Furthermore, researchers have revealed that an increase in cortisol after acute psychological stress is associated with less attentional bias for threat words in a dot probe task^4^. Helfinstein, White, Bar-Haim, and Fox (2008) found that socially anxious individuals showed an attentional bias toward angry faces after a neutral prime, but no attentional bias was found after a threatening prime^5^. However, these studies have solely focused on behavioral outcomes; thus, the exact stage of information processing in which these effects operate remains unknown.

Event-related potentials (ERPs) can be used to distinguish the neural sub-processes that are involved in complex cognitive functions. To our knowledge, only one study has examined the effects of acute stress on attentional bias using ERPs^6^. In this study, social drinkers performed an oddball task under acute stress or control conditions. The results showed that there was an increased P300 for target alcohol-related images compared to neutral distracters in the control condition, and this effect disappeared under stress. However, it still unclear how acute stress affects the distinct cognitive steps in attentional bias in healthy populations.

The dot probe task is frequently used to measure attentional bias and has received considerable empirical support as an index of attentional bias for threat^7–9^. In the dot probe task, participants are instructed to respond to a target when it replaces one of two cue locations that are simultaneously presented^9^. Attention to emotional stimuli can be assessed using emotional facial expressions as stimuli in a dot probe task^10^. In this task, a faster response to the targets that replace a negative stimulus compared to the targets that replace a neutral stimulus suggests attentional bias toward threatening stimuli^9^. In addition, research has reported greater accuracy to targets that appear in the same location as negative stimuli compared to neutral stimuli in dot probe attention tasks. Such a response pattern is considered an indication of enhanced attention to the location of negative stimuli^7^, which may also suggest an attentional bias toward threat-inducing stimuli.

Performance on the dot probe task entails two different stages: (a) processing the emotional cues and (b) processing the subsequent targets^11^. In the cue presentation stage, the P100 peaks at approximately 100 ms after the stimulus is presented and reflects the processing of low-level stimulus features and the mobilization of automatic attentional resources^12^. The N170 is a face perception component that occurs approximately 170 ms after the face is presented, with the largest peak amplitude appearing at lateral posterior electrode sites^13^. When processing subsequent targets, most researchers agree that the attentional bias to threat primarily manifests in the P100 component in response to the target^10,14,15^. These studies have consistently reported an increased P100 amplitude for targets replacing threat stimuli compared to targets replacing neutral stimuli, suggesting an enhanced attention to threats. Previous studies using the cue-target paradigm have also revealed enhanced contralateral attention-related modulation in the target-evoked occipital P100 component^16,17^. In addition, targets elicit a P300 component, which indicates the allocation of attention resources^18^. Furthermore, the finding that the larger ERP (C1) amplitude evoked by the face pair in the cue stage and the larger validity effect on P100 evoked by the target was reported^14^, suggesting potential associations between the processing of the cue and the target.

This study aimed to investigate the effects of acute psychological stress on attentional bias to threat-related stimuli using both ERP and behavioral methods in healthy participants. Acute psychological stress was induced using a modified Trier Social Stress Task (TSST)^19,20^. When faced with acute stress, two systems, the autonomic nervous system (ANS) and hypothalamo-pituitary-adrenocortical (HPA) axis become activated^21^. Further, stress is accompanied by negative affect^22^. The ANS provides the most immediate response to stressor exposure, causing rapidly increased heart rate (HR) and blood pressure within seconds. Cortisol is the final effector of the HPA axis; the activation of the HPA axis is relatively slow and requires several minutes to reach peak levels^21^. Thus, HR and subjective emotional state were used to evaluate the fast reactions to stress and cortisol was used to evaluate the slow reaction to stress. In addition, researchers have found considerable individual differences in cortisol response to acute stress^23,24^. In two studies, low cortisol responders became avoidant and high cortisol responders became vigilant to angry faces^25,26^. The present study examined the relation of stress and attentional bias toward threat, thus, stressed participants were split *post hoc* into Low and High cortisol responders according to the median of cortisol increase induced by stress^27^. Attentional bias was measured with a modified dot probe task. According to previous behavioral studies, we expected that attentional bias to threat would be suppressed under acute stress which was measured via behavioral and ERP components. Furthermore, considering the potential links between the processing of the cue and target^14^, we were also interested in how these associations were altered under acute stress.

## Results

### Self-Report Results

**Figure 1:**
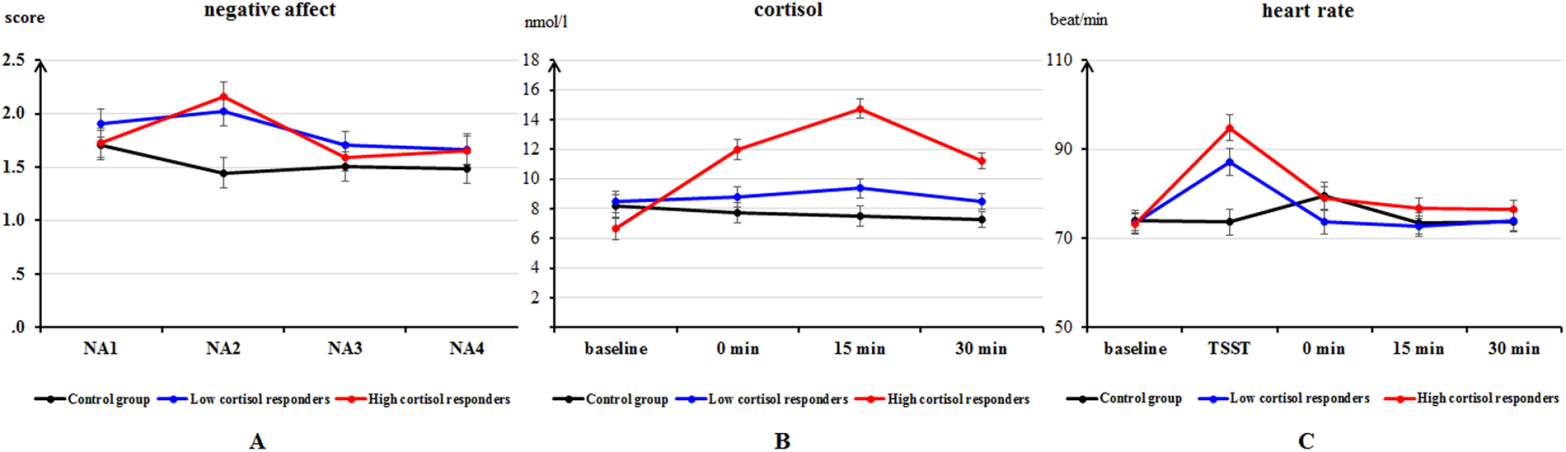
Means and standard errors for negative affect (**A**), cortisol (**B**) and heart rate (**C**) over time for the Control group, the Low and High cortisol responders.

No significant Group difference was found for trait anxiety (*p* > 0.05). For state anxiety, there was a significant main effect of Time, *F* (1, 53) = 606.87, *p* < 0.001, η_*P*_^2^ = 0.92, indicating that participants had a higher state anxiety score at the end of the experiment compared with the baseline measurement. The Group differences and the interaction of Group × Time were nonsignificant. For negative affect, there was a significant main effect of Time, *F* (2.35, 124.27) = 7.88, *p* < 0.001, η_*P*_^2^ = 0.13, and a Group × Time interaction, *F* (6, 159) = 3.69, *p* < 0.01, η_*P*_^2^ = 0.12. The LSD post-hoc tests revealed that both the Low and High cortisol responders had higher scores on negative affect than the Control group immediately after the stress induction (*ps* < 0.01). The Group differences were not significant at other time points (see Fig. 1A). For positive affect, a main effect of Time was significant, *F* (2.23, 118.41) = 10.25, *p* < 0.001, η_*P*_^2^ = 0.16. Participants had higher positive affect score at the baseline than at 0 min (*p* < 0.10), 15 min (*p* < 0.01) and 30 min (*p* < 0.001) and higher positive scores were also found at 0 min compared to 30 min post-treatment (*p* < 0.05). The main effect of Group and Group × Time interaction was nonsignificant.

### Physiological Measurement Results

#### Cortisol Results

The ANOVA for cortisol levels revealed significant main effects of Group, *F* (2, 53) = 11.60, *p* < 0.001, η_*P*_^2^ = 0.31, and Time, *F* (2.51, 132.91) = 18.35, *p* < 0.001, η_*P*_^2^ = 0.26. There was also a significant Group × Time interaction, *F* (6, 159) = 17.27, *p* < 0.001, η_*P*_^2^ = 0.40. The LSD post-hoc tests revealed that (1) the group differences were nonsignificant at baseline; (2) the High cortisol responders had higher cortisol levels than the Control group (*p* < 0.001) and the Low cortisol responders (*p* < 0.01) at 0 min post-stress induction, while the difference between the latter two groups was nonsignificant; (3) the High cortisol responders had a higher cortisol levels than the Low cortisol responders (*ps* < 0.01), that was, in turn, higher than the Control group at 15 min and 30 min post-stress induction (*p*_1_ = 0.05, *p*_2_ = 0.10; see Fig. 1B).

#### Heart rate results

The ANOVA indicated that the main effect of Time, *F* (2.76, 146.17) = 67.55, *p* < 0.001, η_*P*_^2^ = 0.56, and the Group × Time interaction were significant, *F* (8, 212) = 6.47, *p* < 0.001, η_*P*_^2^ = 0.20. Simple effects analysis revealed that the High cortisol responders had higher heart rates than the Control group only during the stress induction (*p* < 0.001), and the differences between Low cortisol responders and the Control group were nonsignificant (see Fig. 1C).

#### Behavioral Results

The ANOVA results showed that no significant main effects of Group, Cue Validity, or Visual Field, nor were there significant interactions related to these three variables on RTs and accuracy on go trials.

**Figure 2:**
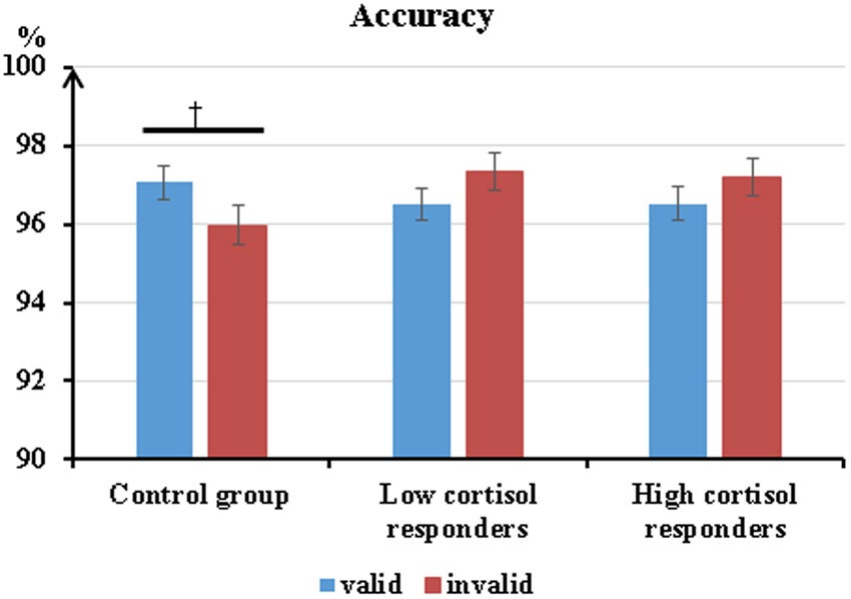
Mean accuracies and standard errors for the Control group, the Low and High cortisol responders in valid and invalid nogo trials (reflects the correct percentage of non-responses on these trials). Valid: when the target replaces an angry faces; Invalid: when the target replaces a neutral faces. ^†^*p* = 0.07.

For the nogo trials, the results showed a Group × Cue Validity interaction on the correct rejection rate, *F* (2, 53) = 3.49, *p* < 0.05, η_*P*_^2^ = 0.12. Post-hoc tests revealed that the Control group showed marginally higher accuracy in valid trials (M ± SE: 97.06 ± 0.42%) than invalid trials (M ± SE: = 95.97 ± 0.49%; *p* = 0.07), while this trend was not found in the Low and High cortisol responders (see Fig. 2). The main effects of Group, Cue Validity and Visual Field as well as other interactions related to the three variables were nonsignificant.

#### ERP Results

ERPs to the face pair.

*P100*. Neither the main effects of Group, Laterality, nor their interaction significantly affected P100 amplitudes or latencies (all *ps* > 0.05).

**Figure 3:**
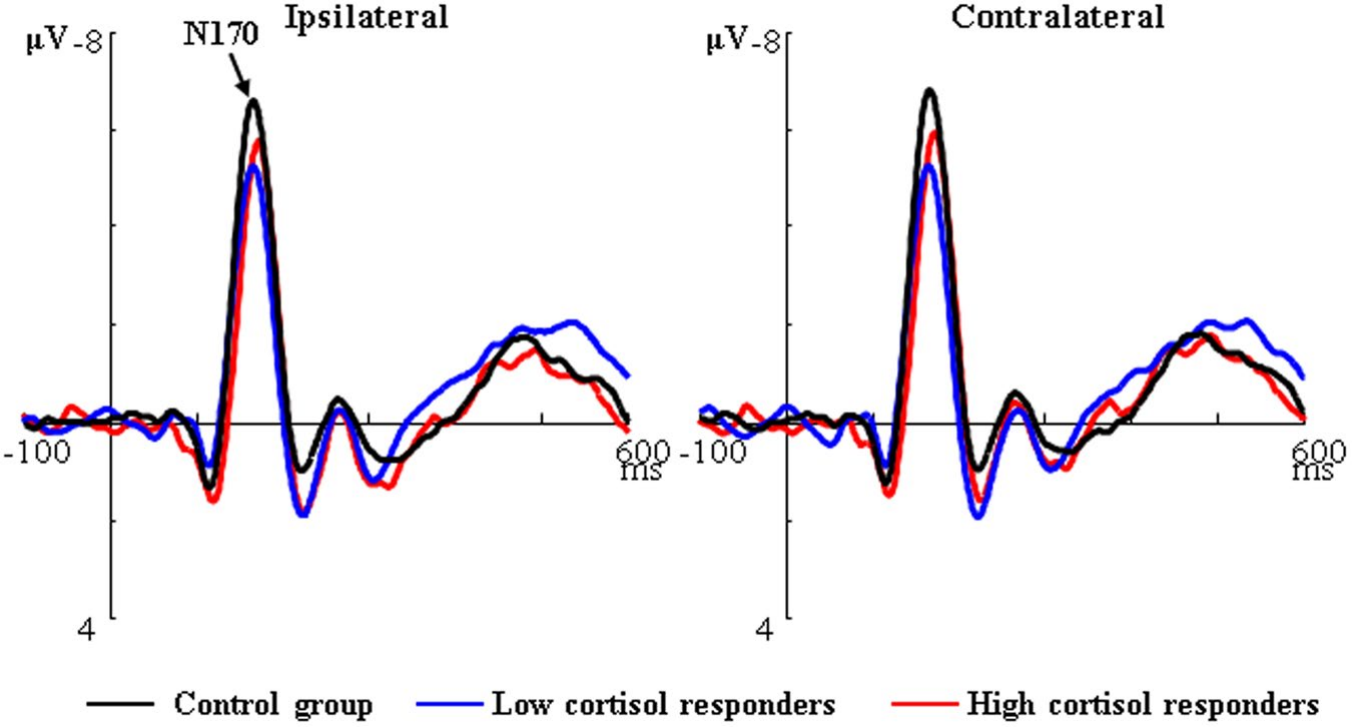
The ipsilateral/contralateral N170 based on pooled activity recorded at PO5/PO7-PO6/PO8 sites, time-locked to the onset of the face pair for the Control group (black line), the Low (blue line) and High cortisol responders (red line). Ipsilateral/Contralateral: electrodes ipsilateral/contralateral to the location of the emotional face.

*N170*. The results revealed that the Group × Laterality interaction for N170 amplitudes was significant, *F* (2, 53) = 3.78, *p* < 0.05, η_*P*_^2^ = 0.13. Post-hoc tests showed that the Control group only had marginally greater N170 than the Low cortisol responders (*ps* = 0.05) and the difference between the Control group and the High cortisol responders was nonsignificant (ipsilateral: *p* = 0.10; contralateral: *p* = 0.17; see Fig. 3). The main effects of Group and Laterality on N170 amplitudes were nonsignificant. For N170 latencies, we did not find any significant main effects of Group or Laterality or their interaction.

#### ERPs to the target

P100 amplitudes. No significant main effects of Group, Cue Validity or Laterality, nor interactions related to these variables were noted.

**Figure 4:**
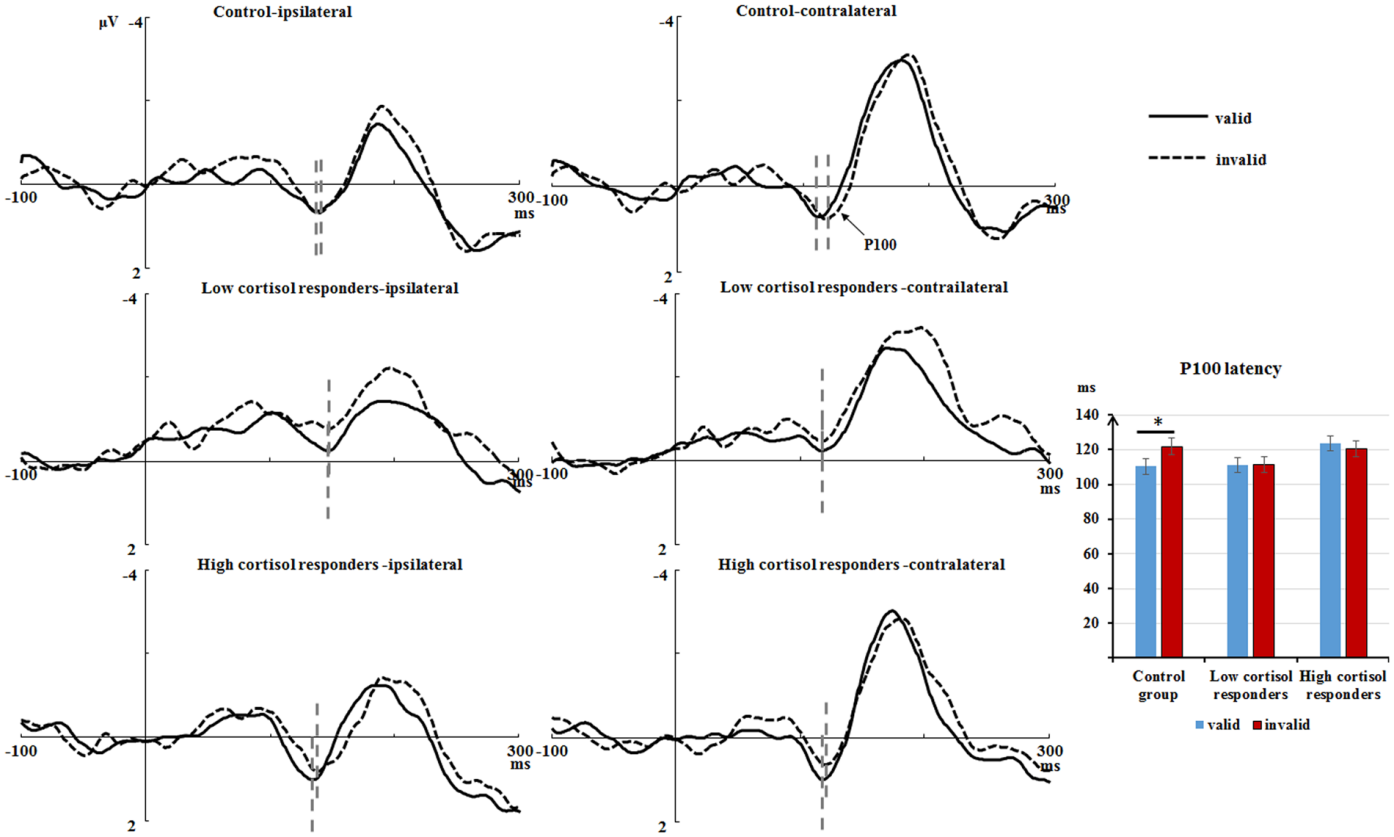
Left panel: Ipsilateral and contralateral waveforms based on pooled activity recorded at CB1/O1-CB2/O2 sites, time-locked to the onset of targets in the valid (solid line) and invalid conditions (dashed line) for the three groups; Ipsilateral/Contralateral: electrodes ipsilateral/ contralateral to the location of the target; Right panel: peak latencies of the target-evoked P1 components in the valid and invalid conditions; *p < 0.05.

P100 latencies. There was a marginally significant Group × Cue Validity interaction for P100 latencies, *F* (2, 53) = 2.92, *p* = 0.06, η_*P*_^2^ = 0.10. Further analysis showed that the P100 peaked earlier in the valid trials (M ± SE: 110.31 ± 4.39 ms) compared to the invalid trials (M ± SE: 121.84 ± 4.80 ms) in the Control group, *F* (1, 53) = 6.60, *p* < 0.05, η_*P*_^2^ = 0.11, however, this effect did not occur in the Low and High cortisol responders (see Fig. 4). The main effect of Laterality was significant, *F* (1, 53) = 23.96, *p* < 0.001, η_*P*_^2^ = 0.31. The contralateral P100 peaked earlier (M ± SE: 110.36 ± 2.50 ms) than the ipsilateral P100 (M ± SE: 122.54 ± 2.68 ms). No other significant effects were noted.

**Figure 5:**
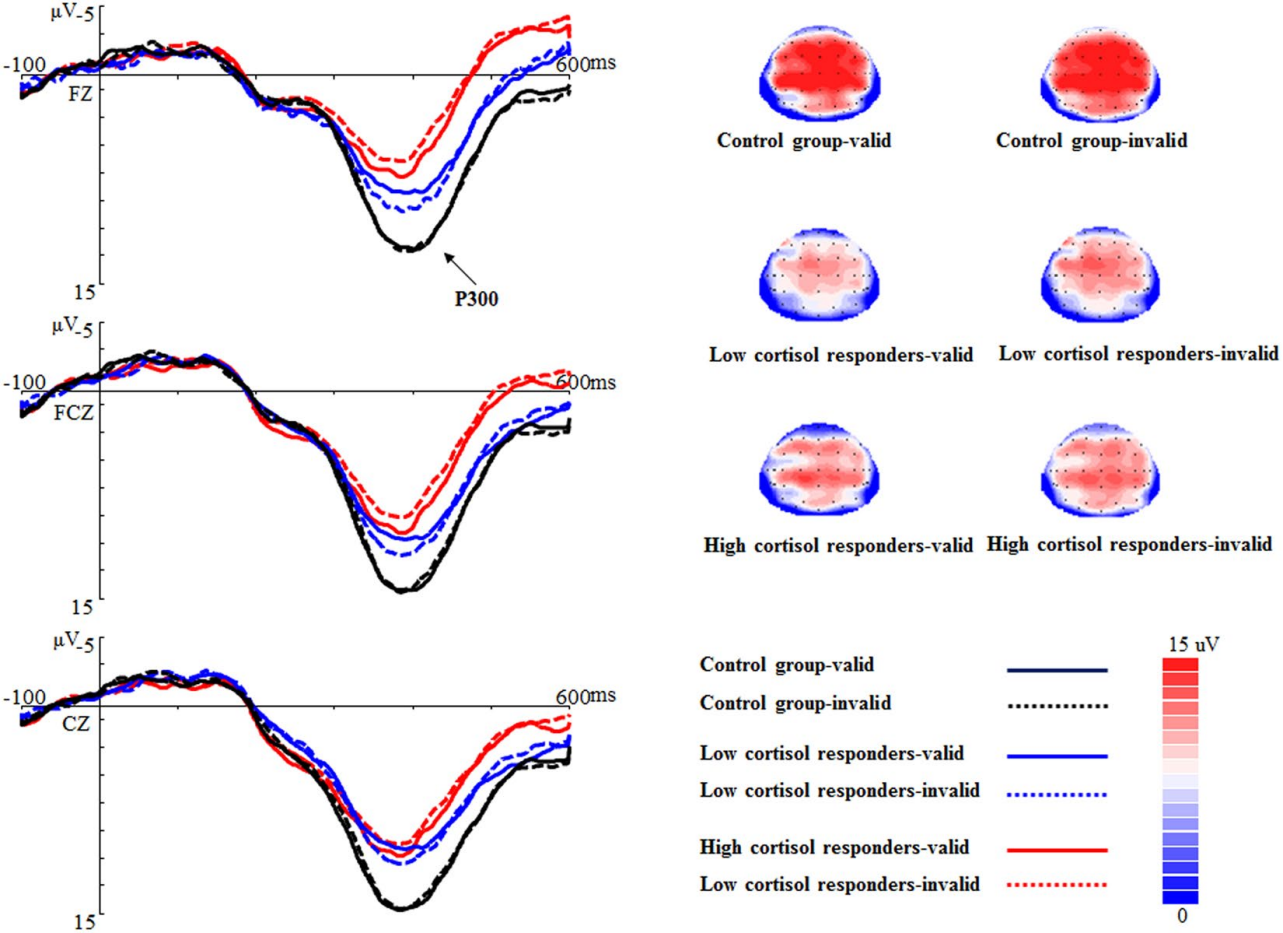
The P300 component (FZ, FCZ and CZ) and topographic maps (CZ) time-locked to the target onset for the Control group, the Low and High cortisol responders in the valid and invalid conditions. Valid: when the target replaced an angry faces; Invalid: when the target replaced a neutral faces.

*P300*. The main effect of Group was significant, *F* (2, 53) = 5.01, *p* = 0.01, η_*P*_^2^ = 0.16. Post-hoc tests showed that the Control group (M ± SE: 15.80 ± 1.19 μv) had larger P300 amplitudes than the Low (M ± SE: 11.75 ± 1.15 μv, *p* = 0.05) and High cortisol responders (M ± SE: 10.87 ± 1.15 μv, *p* < 0.05). The differences between the latter two groups were nonsignificant. There was a marginally significant Group × Cue Validity interaction for P300 amplitudes, *F* (2, 53) = 2.63, *p* = 0.08, η_*P*_^2^ = 0.09. Further analysis revealed that this interaction was mainly driven by the main effect of Group (see Fig. 5). No other significant main effects or interactions for P300 amplitudes were found. The main effects of Group and Cue Validity and interactions between the two variables were nonsignificant for P300 latencies.

#### Correlation Analyses

The ipsilateral and contralateral face-N170 amplitudes were positively correlated with the contralateral-invalid target-P100 latencies (*r*_*ipsi*_ = 0.65, 95% confidence interval: 0.34 ~ 0.85; *r*_*contra*_ = 0.67, 95% confidence interval: 0.39 ~ 0.85) and negatively correlated with P300 amplitudes (valid: *r*_*ipsi*_ = −0.45, 95%, confidence interval: −0.71 ~ −0.07; *r*_*contra*_ = −0.43, 95% confidence interval: −0.69 ~ −0.05; invalid: *r*_*ipsi*_ = −0.51, 95% confidence interval: −0.74 ~ −0.20; *r*_*contra*_ = −0.50, 95% confidence interval: −0.73 ~ −0.16) in the Control group. These results suggest that the larger the N170 amplitude as induced by the face pair, the earlier the contralateral-invalid P100 peak and the larger the P300 amplitudes as induced by the target in the Control group. However, these effects were absent in the Low and High cortisol responders.

## Discussion

The present study investigated the influence of acute psychosocial stress on attentional bias to threatening stimuli using behavioral and electrophysiological measures. The acute stress response was successfully evoked, as indicated by the higher negative affect, increased heart rate, and higher cortisol levels in the stress group compared to the Control group. The behavioral results indicated that the Control group showed a trend of more accurate responses to the valid compared to the invalid trials, while this effect was not seen in the stress group (neither Low nor High cortisol responders). The ERP data showed that the P100 component induced by the target peaked earlier in the valid compared to the invalid trials in the Control group. However, this effect was absent in both the Low and High cortisol responders of the stress group. In addition, we found reduced P300 amplitudes at the target onset in the stress group relative to the control group.

The control group showed marginally higher accuracy on the valid nogo trials than invalid nogo trials, which may be a product of enhanced attention to the location of angry faces^7^, indicating an attentional bias effect. However, acute stress interfered with the attentional resources necessary to diverting from angry faces and resulted in no attentional bias effect in the Low and High cortisol responders of the stress group. These results support our hypothesis and are similar to previous reports of altered attention under stress^2,3^, although these studies reported that the effect was on the response time to the negative stimuli, rather than on the accuracy of non-response, as in the current study.

More importantly, the present study found that the Control group showed earlier P100 latencies on valid than invalid trials, but this effect was not found in either the Low and High cortisol responders of the stress group. The P100 component reveals the mobilization of automatic attentional resources^12^, and its latency reflects the speed of perceptual processing^28^. A shorter P100 latency indicates a faster processing speed of the target that replaces the angry face and suggests a normal, adaptive attentional bias toward the threatening stimulus. However, this difference was not seen under acute stress, and it was not related to the stress level; that is, attention was equally allocated to angry and neutral faces in stressed participants, indicating a stress-induced suppression of the attentional bias. These results were similar to the behavioral results.

This finding could be further evidence supporting the idea that attention orientation to threat is suppressed under stress. These results provide neural evidence for how attentional bias may develop under acute stress. One possibility for the reduced threat bias under stress may be that acute stress shifts the processing priorities from the less threatening stimulus (i.e., angry faces) to the more threatening stimulus (i.e., acute stress)^5,29^. In this case, lower accuracy or longer response time may be found in the stress group. However, our results did not support this view as relatively higher accuracy was found in the Low cortisol responders and High cortisol responders (though to a lesser degree in the low cortisol responders) compared to the Control group. Another possibility may be that acute stress makes it difficult to distinguish threat from non-threat information. Shackman, Maxwell, McMenamin, Greischar, & Davidson (2011) reported that threat of shock can increase neural responses in extrastriate visual cortex, even for neutral visual stimuli^30^. Acute stress can also shift amygdala function toward heightened sensitivity with lower levels of specificity, that is, stress augments amygdala responses to both threat-related stimuli and non-threatening stimuli, thereby diminishing a threat selective response pattern under stress^31^. According to this view, higher accuracy or shorter response time may be found in the stress group. Indeed, our results support this view. However, given the marginally significant results, the conclusion should be treated cautiously and more studies are needed to further test these implications.

Some studies that have assessed stress effects on attention have produced inconsistent results^25,32^, in which attentional bias to threat was enhanced in an acute stress condition compared to a control condition. There may be several reasons for these mixed results. One possibility may be different cue presentation times. Mogg *et al*.^32^ used a dot probe task with a 500-ms cue presentation time^32^, while the present study adopted a modified dot probe task with a 100-ms cue presentation time. Previous research observed that attention orienting to threats typically occurred when the stimuli were presented for a short duration (less than 100 ms)^8,33^. Attention disengagement from threats tends to occur when the duration is much longer (500 ms or longer), which is sufficient time for attention to shift one or more times^33^. Using different task paradigms may be another contributing factor to the inconsistent results reported in the literature. Roelofs *et al*.^25^ used a masked emotional Stroop task to show that those who produce the largest stress cortisol responses also showed greater vigilance toward angry faces^25^. The current results from a dot probe task do not show such a pattern. Some researchers have argued that the emotional Stroop task indirectly measures attentional bias and that a longer response time to threat-related information may reflect a response bias rather than an attentional bias^34,35^. Finally, gender may also be a potential moderator. In the research by Roelofs *et al*.^25^ and Mogg *et al*.^32^, both men and women were included, while only men were included in the present study. Previous studies have revealed that compared to young women, young men have higher cortisol responses after exposure to acute stress^36^, which could make men perceive stressful situations as more threatening, resulting in more conservative, inhibited behavior^37^.

In addition, the Low and High cortisol responders had smaller P300 amplitudes for targets compared to the Control group both in the valid and invalid conditions. The results were consistent with studies in clinical populations (i.e., PTSD and anxiety disorder) who also show reduced P300 amplitudes, suggesting a deficit in the amount of attentional resources allocated during stimulus evaluation and memory updating processes^38,39^. These results may be attributed to the detrimental effect of acute stress on higher-order PFC abilities, i.e., attentional regulation^40,41^. The attentional regulation can switch from slow thoughtful ‘top-down’ control by the PFC to reflexive and rapid emotional responses of the amygdala and related subcortical structures^40^. Consistent with this argument, an ERP study found that stress can amplify earlier extrastriate activity, but disrupt later activity associated with the evaluation of task-relevant information as reflected by P300 amplitude^30^. Hence, the smaller P300 in the stress condition may be associated with reduced prefrontal cortex activity under acute stress indicating less efficient cortical processing.

Interestingly, we also found that the N170 amplitudes elicited by the face cue were positively related to the target induced contralateral-invalid P100 latencies and P300 amplitudes both in the valid and invalid trials in the Control group, while these relationships were absent in the Low and High cortisol responders. The correlations in the Control group suggests that higher neural activity at the structural encoding step of face perception may be associated with faster early sensory processing of the subsequent stimuli and more engagement in attention orientation to the following stimuli. Pourtois *et al*.^14^ reported that a larger C1, as evoked by face pairs, was correlated with a larger validity effect on the P100 amplitude^14^. Because C1 is thought to reflect the initial evoked response in the primary visual cortex^42^, Pourtois *et al*. proposed that the correlation might indicate the important functional significance of C1, which allows for processing of the subsequent target that was presented at the same location as the threat. In the present study, N170, another important face-sensitive component, may also have significant functional implication. However, acute stress may interrupt the link between face perception and subsequent target processing, as reflected by the lack of this relationship in the stress condition, which may indicated one mechanism underlying the reduced attentional bias in stressed individuals, as discussed above.

There are several limitations to the current study. First, as only male participants were included in this study, we cannot expand the findings to females. Second, although we examined how and at which stages acute stress influenced the attentional bias, the time frame during which acute stress affects attentional bias is unknown. More research is needed to examine sex differences and how the duration of acute stress affects attentional bias. Finally, the present study used a between-subjects design without measuring the baseline attentional bias before the stress/control treatments, which may have not fully eliminated possible individual differences between the two groups. Further research should use a pre-post treatment design to eliminate individual differences.

## Conclusion

In summary, our results suggest that acute psychosocial stress impairs attentional bias. The reduced attentional bias on the P100 component and reduced P300 component may suggest that the underlying neural mechanisms that acute stress may lead to indiscriminate selective response to threat and reduced efficiency of cortical processing.

## Methods

### Participants

The study was approved by the Ethics Committee of Human Experimentation at the Institute of Psychology, Chinese Academy of Sciences. All procedures were performed in accordance with relevant guidelines and regulations. Due to sex differences and menstrual cycle effects on the stress response and cognitive functioning^36,43^, only healthy male undergraduates were recruited from universities in Beijing. Participants who met the following criteria were included: (1) no medication that affects the hypothalamic-pituitary-adrenal axis (e.g., corticosteroids) within one month of testing; (2) no past or present neurological or psychiatric diagnoses; (3) no periodontitis or any wounds in the mouth; (4) no cold or other medication within the previous two weeks; (5) no overnight shift work or irregular circadian rhythms; (6) no excessive alcohol (more than two alcoholic drinks daily) or nicotine consumption (more than five cigarettes a day); and (7) normal or corrected-to-normal vision and intact color vision. Participants were first screened for the inclusion criteria and were then informed via telephone to refrain from heavy exercise, eating or drinking, except for water, for two hours prior to arriving in the laboratory. In addition, after arrival, participants were asked to avoid drinking water for a half hour prior to the first saliva collection.

Sixty-two male undergraduate students reporting right-handedness were recruited. All participants provided written informed consent and received financial compensation for participation. In order to explore the potential difference in attentional tasks between the High and Low cortisol responders, the number of participants in the stress condition was nearly twice as many as that in the control condition to ensure equal cell sizes. Participants were randomly assigned to the control or stress conditions. Five participants were excluded due to poor behavioral performance (below 3 standard deviations from the mean) or missing more than three values on cortisol, which resulted in 38 participants in the stress condition and 18 participants in the control condition. The Control group (mean age = 20.67 ± 1.46 years) and the stress group (mean age = 21.08 ± 1.78 years) were matched for age.

### General Procedure

The experiment occurred between 13:30 p.m. and 18:30 p.m. Participants rested for 30 min upon arrival at the laboratory, during which they completed the informed consent document and the State-Trait Anxiety Inventory (STAI)^44,45^. Then, they were prepared for the electroencephalogram (EEG) recording. Next, participants practiced the dot probe task. After that, participants were randomly assigned to perform the TSST or the control task. Then, participants completed the formal dot probe task in a dimly lit, sound-attenuated room. Finally, participants rated their state anxiety level on the STAI state scale (approximately 30 min after the stress or control tasks). Salivary samples, heart rate, and responses on the Positive Affect and Negative Affect Schedule (PANAS)^46,47^ were collected 30 min after arrival as a baseline and at 0 min, 15 min, and 30 min after the end of the stress or control tasks. In addition, heart rate was continuously recorded throughout the stress or control tasks.

### Materials

The face stimuli were from the Japanese and Caucasian Facial Expressions of Emotion (JACFEE) and Neutral Faces (JACNeuF) sets^48^. The properties of the photographs such as contrast and luminance were normalized. Four angry (2 male) and four neutral (2 male) expressions of different Asian actors (totally 8 actors) were used, and the angry and neutral faces did not differ in luminance and contrast. The photographs were presented in pairs with an angry face and a neutral face of different actors with the same gender. There were two different types of face pairs: angry (left) - neutral (right) and neutral (left) - angry (right). The angry face appeared an equal number of times to the left or right of the fixation cross. Each face was enclosed in a black rectangular frame measuring 7 × 10 cm, with a 10.5 cm distance from its center to the white fixation cross. Participants were seated in front of the screen at a viewing distance of 70 cm, which resulted in a visual angle of 8.5° as measured from the fixation cross to the center of each image. The probe was a white upward or downward triangle measuring 2.26 × 2.61 cm (1.85° × 2.14° of visual angle). The probability of the probe appearing at the location of the angry or the neutral face was the same (50%).

### Modified Dot Probe Task

A modified dot probe task that stemmed from Brosch *et al*.’s^10^ study was used with the following two considerations: (a) to investigate covert spatial orientation toward threats without contamination from motor-related activities^14^; and (b) to collect enough behavioral data.

**Figure 6:**
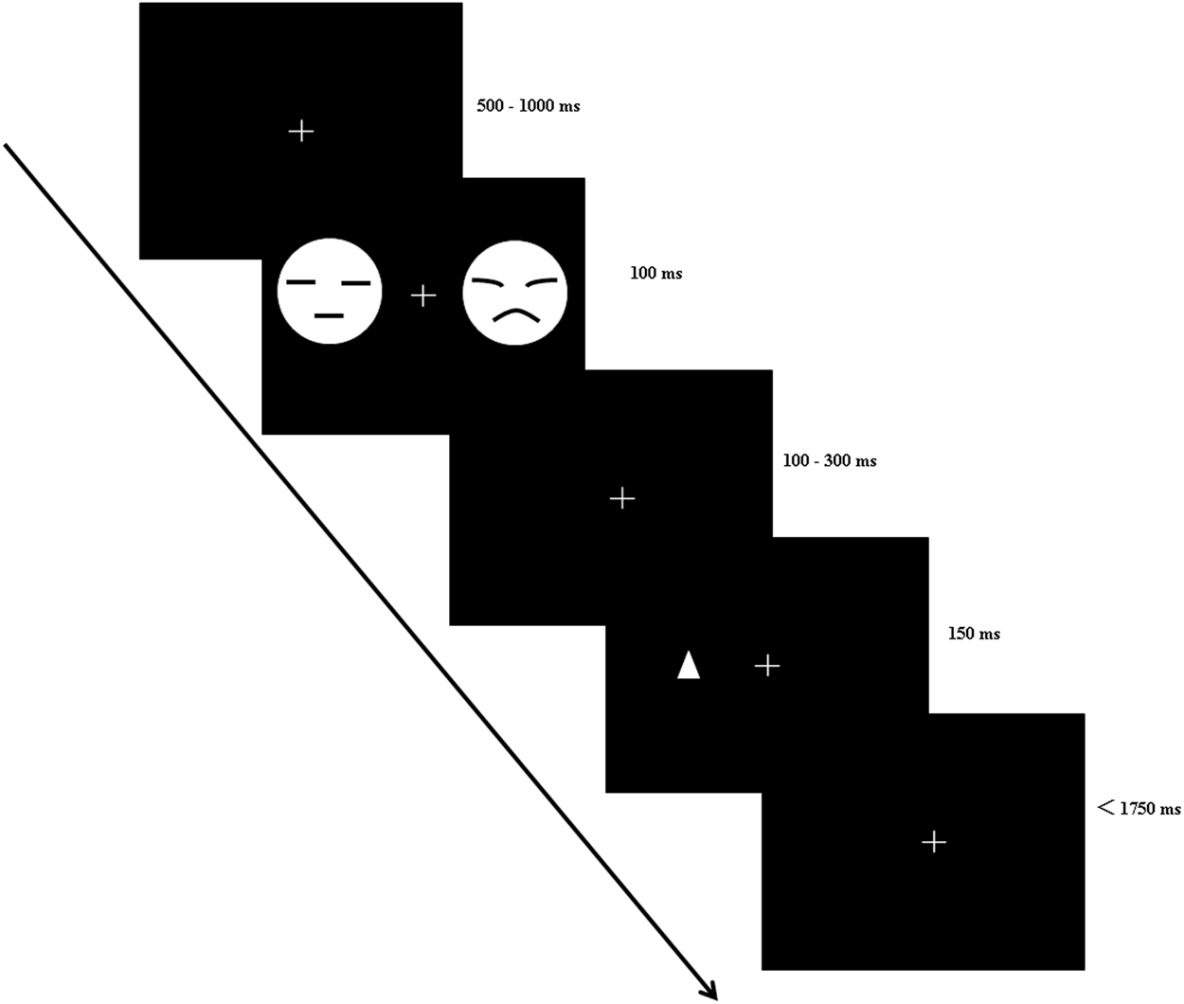
The schematic diagram of the dot probe task. Note: the faces showed here were just a sketch and not the ones used in the formal experiment.

Each trial started with a 2 × 2 cm white fixation cross that was presented for a random duration between 500 and 1,000 ms, which was followed by a pair of faces that were displayed for 100 ms. Then, the fixation cross was presented for a random duration between 100 and 300 ms after the offset of the face pairs. Next, a probe appeared for 150 ms at the previous location of one of the faces. Participants were required to use their right index finger to press a key for the go stimulus (e.g., upward triangle) while withholding the responses to the nogo stimulus (e.g., downward triangle). The maximum response time was 1,900 ms, after which a new trial began (see Fig. 6). In the go trials, the direction of the triangle pointing upward or downward was counterbalanced across participants. The present study included 25% go trials and 75% nogo trials.

The present experiment consisted of two practice blocks with 8 trials each. The formal experiment included four test blocks with 96 trials and a short break between blocks. A valid trial indicates that the target replaced the location of the angry faces, while an invalid trial indicates that the target replaced the location of the neutral faces. Shorter response time for go trials or higher accuracy for go or nogo trials in the valid condition than the invalid condition indicates an attentional bias toward the threat.

### Stress Induction and Control Condition

The present study used a modified TSST, which is more effective in eliciting cortisol responses than the original TSST^19,49^. The modified version consists of similar three phases as the original: preparation (5 min), speech (5 min), and mental arithmetic (5 min). Participants were instructed to imagine that they were accused of shoplifting and had to deliver a speech to defend themselves in front of three store managers. Participants were also informed that their performance would be videotaped and evaluated by the research team. At the end of the speech, participants were instructed to complete a 5-min backward mental arithmetic task (subtracting 13 from 1022) and must start from the beginning if any mistakes were made.

In the control protocol, participants performed a less stressful task, which was similar to the TSST with respect to the time course and physical and mental demands^50,51^. In the preparation phase, participants were asked to read an article about travel and summarize the main contents. Then, they were required to give a speech in front of a video camera. Participants were told that they could refer to their notes and their performance would not be evaluated. Next, participants completed a simple written arithmetic test for 5 min. This protocol was performed in the test room with only the participant in the room.

### Subjective Measures

#### State and trait anxiety

Anxiety was measured with the Chinese version of the STAI^44,45^, which has two subscales: state anxiety and trait anxiety. Each subscale contains 20 items. The test-retest reliabilities for the STAI state scale and STAI trait scale are 0.88 and 0.90, respectively^52^. Participants rated each item on a four-point Likert scale, with a higher score indicating a higher level of anxiety.

#### Positive and negative affect

The PANAS is a 20-item self-rating scale for measuring positive and negative affect, which was developed by Watson *et al*.^47^. This study used the Chinese version^46^, which has a Cronbach’s alpha of 0.82 for the full scale and 0.85 and 0.83 for the positive and negative affect subscales, respectively.

### Physiological Measures

#### Saliva sample collection

Salivette collection devices (Sarstedt, Nümbrecht, Germany) were used to obtain saliva samples, which were measured at four time points: 30 min after arriving in the laboratory as a baseline and 0 min, 15 min, and 30 min after the end of the TSST or control procedure. Participants were required to chew a cotton pledget for 2 min. The samples were stored at −20 °C until they were assayed. An electrochemiluminescence immunoassay (Cobas e601, Roche Diagnostics, Numbrecht, Germany) was used to analyze the cortisol concentration, and the following parameters were used: sensitivity, 0.500 nmol/L (lower limit), and standard range in assay, 0.5–1750 nmol/L. Intra- and inter-assay variations were less than 10%. Five participants, who were missing three or more samples due to insufficient saliva, were excluded from the final analysis. Participants missing one or two cortisol values were included. Missing baseline values were substituted with the average cortisol concentration of all participants at that time point, and missing values at other time points were substituted with the average cortisol separately for each group^53^. The number of final participants for the cortisol analysis was 56 (the Control group *N* = 18, the stress group *N* = 38). Participants in the stress condition were divided into the Low cortisol responders (*N* = 19) and High cortisol responders (*N* = 19) according to a median split (3.80 nmol/l) of cortisol change, which was computed by subtracting baseline cortisol level from peak-time cortisol level (15 min post-stress induction in this experiment)^27^.

#### Heart rate recording

Heart rate (HR; beats per min) was measured using the electrocardiogram (ECG) amplifier module from BIOPAC MP150 for Windows (BIOPAC Systems, Inc., CA, USA). ECG was measured via three Ag–AgCl disposable electrodes, which were placed on the right carotid artery and the left and right medial malleolus after the surface was cleaned with alcohol. Except for the continuous recording during the TSST/control task, HR was collected at the same time as each saliva collection for a 5 min. However, 15 min post-TSST/control task, the ECG lasted only for 2 min to ensure that the attentional bias task was completed during the period of cortisol elevation in response to the TSST. HR was averaged at each assessment point for each participant offline.

### EEG Recording and Preprocessing

During the dot probe task, the EEG was recorded from 64 channels using Ag/AgCl electrodes in a cap conforming to the international 10–20 system (Neuroscan Inc., Charlotte, North Carolina, USA), with an on-line reference to the left mastoid and an off-line algebraic re-reference to the average of the left and right mastoids. The electrooculogram (EOG) was recorded by placing electrodes at the outer canthus of each eye as well as above and below the left eye. Impedance was maintained at less than 5 kΩ. Signals were amplified with a 0.05–100 Hz bandpass filter and digitized at 1,000 Hz.

Scan 4.5 software (Neuroscan, USA) was used to process the EEG data. Ocular artifacts were removed from the EEG signal with a regression procedure that was implemented in the Neuroscan software^54^. Data were digitally filtered using a low-pass filter of 30 Hz. An interval of 100 ms before the face and target presentations was used as the baseline for the face and target ERPs. Epochs were created around the face and target onsets (from −100 ms to +600 ms) and epochs with amplitudes that exceeded ±100 µV were discarded. After epoching, baseline corrections were performed. Finally, the trials that were in the same condition (including the face pair and target onset-locked ERPs) were averaged, and correct rejections of the nogo trials were used to create the ERPs. Overall, for N170 evoked by the face pair, the number of trials for each condition was 144 and less than 36.5% of the trials were rejected in the three groups (Control group, Low and High cortisol responders) because of artifacts and outliers in reaction times. For P1 evoked by the target, the number of trials for each condition was 72 and less than 43.1% trials were rejected in the three groups. For P3 evoked by the target, the number of trials for each condition was 144 and less than 30.6% trials were rejected in the three groups.

### Data Analysis

One-way analysis of variances (ANOVAs) was used to examine the group differences on trait anxiety levels. Repeated measures ANOVAs were performed on state anxiety levels, positive and negative affect, cortisol levels, and HR, with Measurement Time Points as within-subjects factors and Group (Control group, Low and High cortisol responders) as a between-subjects factor.

All trials with incorrect responses and trials with response time faster than 100 ms or slower than 1500 ms were excluded from behavioral and ERP analyses. For behavioral data, a repeated measures ANOVA was performed on response time (RT) for the go trials with Group as a between-subjects factor and Cue Validity (valid, invalid) and Visual Field (left, right) as within-subjects factors. Similar ANOVAs were also performed on the percentage of hits in the go trials and the rate of correct rejections in the nogo trials.

For ERPs evoked by the face pairs, the P100 and N170 components were analyzed. The P100 and N170 peak amplitudes and latencies were measured at occipital sites (O1/2, CB1/2; time window: 60–160 ms) and bilateral occipital sites (PO5/6, PO7/8; time window: 130–210 ms) separately. Repeated measures ANOVAs were performed on the P100 and N170 amplitudes/latencies separately with Group as a between**-**subjects factor and Contralaterality (ipsilateral vs. contralateral to the location of the angry face) as a within**-**subjects factor. The ipsilateral waveform was calculated by averaging the left-sided electrodes to the left-sided angry face and the right-sided electrode to the right-sided angry face, and the contralateral waveform was calculated by averaging the left-sided electrodes to the right-sided angry face and the right-sided electrodes to the left-sided angry face.

For ERPs evoked by the target onset, we analyzed P100 and P300 components. P100 peak amplitudes and latencies were measured across the O1/2 and CB1/2 sites (time window: 60–180 ms). Repeated measures ANOVA with Group as a between-subjects factor and Cue Validity and Contralaterality (electrode ipsilateral vs. contralateral to the location of the target) as within-subjects factors. For the target-evoked P100, the ipsilateral and contralateral waveforms were calculated in a similar way as for the face pairs according to the location of the target. The P300 component was measured at midline sites Fz, FCz, and Cz (time window: 250–600 ms), and the visual field factor was not included in the final analysis as it did not interact with group or cue validity (*ps* > 0.46). The repeated measures ANOVA was performed on the P300 component with Group as a between-subjects factor and Cue Validity and Site as within-subjects factors. Greenhouse-Geisser correction was used to correct the degrees of freedom and Bonferroni corrections were applied to correct the alpha levels. Interaction tests were conducted using the least significant difference (LSD) method.

To explore the potential correlations between face perception during the cue stage and attentional bias during the target response stage, correlation analyses were conducted for the component that was evoked by the face pair (ipsilateral and contralateral N170 amplitudes) and the components that were evoked by targets (ipsilateral and contralateral P100 latencies and the averaged P300 amplitudes at the midline electrodes in the valid and invalid conditions) for the Control, Low and High cortisol responders, separately. To reduce type I error rate on exploratory correlation analyses, the bootstrapping (number of samples: 1000) were performed and 95% confidence intervals surrounding each correlation coefficient are reported.

## References

[1] Putwain DW, Langdale HC, Woods KA, Nicholson LJ (2011). Developing and piloting a dot-probe measure of attentional bias for test anxiety. Learn. Individ. Differ..

[2] Mansell W, Clark DM, Ehlers A, Chen YP (1999). Social anxiety and attention away from emotional faces. Cognition Emotion.

[3] Bakvis P (2009). Trauma, stress, and preconscious threat processing in patients with psychogenic nonepileptic seizures. Epilepsia.

[4] McHugh RK, Behar E, Gutner CA, Geem D, Otto MW (2010). Cortisol, stress, and attentional bias toward threat. Anxiety Stress Coping.

[5] Helfinstein SM, White LK, Bar-Haim Y, Fox NA (2008). Affective primes suppress attention bias to threat in socially anxious individuals. Behav Res Ther..

[6] Ceballos NA, Giuliano RJ, Wicha NYY, Graham R (2012). Acute Stress and Event-Related Potential Correlates of Attention to Alcohol Images in Social Drinkers. J. Stud. Alcohol Drugs..

[7] Carlson JM, Reinke KS (2008). Masked fearful faces modulate the orienting of covert spatial attention. Emotion.

[8] Koster EHW, Verschuere B, Crombez G, Van Damme S (2005). Time-course of attention for threatening pictures in high and low trait anxiety. Behav Res Ther..

[9] MacLeod C, Mathews A, Tata P (1986). Attentional bias in emotional disorders. J. Abnorm. Psychol..

[10] Brosch T, Sander D, Pourtois G, Scherer KR (2008). Beyond fear: rapid spatial orienting toward positive emotional stimuli. Psychol. Sci..

[11] Eldar S, Yankelevitch R, Lamy D, Bar-Haim Y (2010). Enhanced neural reactivity and selective attention to threat in anxiety. Biol. Psychol..

[12] Carretie L, Hinojosa JA, Martin-Loeches M, Mercado F, Tapia M (2004). Automatic attention to emotional stimuli: neural correlates. Hum. Brain Mapp..

[13] Bentin S, Allison T, Puce A, Perez E, McCarthy G (1996). Electrophysiological studies of face perception in humans. J. Cognitive Neurosci..

[14] Pourtois G, Grandjean D, Sander D, Vuilleumier P (2004). Electrophysiological correlates of rapid spatial orienting towards fearful faces. Cereb. Cortex.

[15] Santesso DL (2008). Electrophysiological correlates of spatial orienting towards angry faces: a source localization study. Neuropsychologia.

[16] Fu S, Caggiano DM, Greenwood PM, Parasuraman R (2005). Event-related potentials reveal dissociable mechanisms for orienting and focusing visuospatial attention. Cognitive Brain Res.

[17] Natale E, Marzi CA, Girelli M, Pavone EF, Pollmann S (2006). ERP and fMRI correlates of endogenous and exogenous focusing of visual-spatial attention. Eur J Neurosci.

[18] Polich, J. Theoretical Overview of P3a and P3b in *Detection of change: event*-*related potential and fMRI findings* (ed. Polich, J.) 83–98 (Kluwer, 2003).

[19] Buchanan TW, Bagley SL, Stansfield RB, Preston SD (2012). The empathic, physiological resonance of stress. Soc. Neurosci..

[20] Kirschbaum C, Pirke KM, Hellhammer DH (1993). The Trier Social Stress Test - a Tool for Investigating Psychobiological Stress Responses in a Laboratory Setting. Neuropsychobiology.

[21] Ulrich-Lai YM, Herman JP (2009). Neural regulation of endocrine and autonomic stress responses. Nat Rev Neurosci.

[22] Dickerson SS, Kemeny ME (2004). Acute stressors and cortisol responses: A theoretical integration and synthesis of laboratory research. Psychol Bull.

[23] Kirschbaum C (1995). Persistent high cortisol responses to repeated psychological stress in a subpopulation of healthy men. Psychosom Med.

[24] Kudielka BM, Hellhammer DH, Wust S (2009). Why do we respond so differently? Reviewing determinants of human salivary cortisol responses to challenge. Psychoneuroendocrinology.

[25] Roelofs K, Bakvis P, Hermans EJ, van Pelt J, van Honk J (2007). The effects of social stress and cortisol responses on the preconscious selective attention to social threat. Biol Psychol.

[26] Roelofs K, Elzinga BM, Rotteveel M (2005). The effects of stress-induced cortisol responses on approach-avoidance behavior. Psychoneuroendocrinology.

[27] Dierolf, A. M. Fechtner, J. R. Bohnke, O. T., & Wolf, E. N. Influence of acute stress on response inhibition in healthy men: An ERP study. *Psychophysiology* (2017). 10.1111/psyp.12826 28168718

[28] Thomas SJ, Gonsalvez CJ, Johnstone SJ (2013). Neural time course of threat-related attentional bias and interference in panic and obsessive-compulsive disorders. Biol. Psychol..

[29] Mathews A, Mackintosh B (1998). A cognitive model of selective processing in anxiety. Cognitive Ther. Res..

[30] Shackman AJ, Maxwell JS, McMenamin BW, Greischar LL, Davidson RJ (2011). Stress Potentiates Early and Attenuates Late Stages of Visual Processing. J Neurosci.

[31] van Marle HJF, Hermans EJ, Qin S, Fernández G (2009). From Specificity to Sensitivity: How Acute Stress Affects Amygdala Processing of Biologically Salient Stimuli. Biol Psychiat.

[32] Mogg K, Mathews A, Bird C, Macgregor-Morris R (1990). Effects of stress and anxiety on the processing of threat stimuli. J. Pers. Soc. Psychol..

[33] Cooper RM, Langton SR (2006). Attentional bias to angry faces using the dot-probe task? It depends when you look for it. Behav Res Ther..

[34] Mogg K, Bradley BP, Hallowell N (1994). Attentional bias to threat: Roles of trait anxiety, stressful events, and awareness. Q. J. Exp. Psychol..

[35] Morrison R, O’Connor RC (2008). The role of rumination, attentional biases and stress in psychological distress. Brit. J. Psychol..

[36] Kudielka BM, Kirschbaum C (2005). Sex differences in HPA axis responses to stress: a review. Biol. Psychol..

[37] Granger DA, Weisz JR, McCracken JT, Ikeda SC, Douglas P (1996). Reciprocal influences among adrenocortical activation, psychosocial processes, and the behavioral adjustment of clinic - referred children. Child Dev.

[38] Metzger LJ, Orr SP, Lasko NB, Berry NJ, Pitman RK (1997). Evidence for diminished P3 amplitudes in PTSD. Ann N Y Acad Sci.

[39] Polich J, Herbst KL (2000). P300 as a clinical assay: rationale, evaluation, and findings. Int J Psychophysiol.

[40] Arnsten AF (2009). Stress signalling pathways that impair prefrontal cortex structure and function. Nat Rev Neurosci.

[41] Plessow, F., Fischer, R., Kirschbaum, C. & Goschke, T. Inflexibly Focused under Stress: Acute Psychosocial Stress Increases Shielding of Action Goals at the Expense of Reduced Cognitive Flexibility with Increasing Time Lag to the Stressor. *J Cognitive Neurosci* **23**, 3218–3227 (2011). 10.1162/jocn_a_00024 21452940

[42] Clark VP, Hillyard SA (1996). Spatial selective attention affects early extrastriate but not striate components of the visual evoked potential. J. Cognitive Neurosci..

[43] Kirschbaum C, Kudielka BM, Gaab J, Schommer NC, Hellhammer DH (1999). Impact of gender, menstrual cycle phase, and oral contraceptives on the activity of the hypothalamus-pituitary-adrenal axis. Psychosomatic. Med..

[44] Shen, Y. C. *Psychiatry* 243–263 (People’s Medical Publishing House, 1988).

[45] Spielberger, C. D. & Gorsuch, R. L. Manual for the state-trait anxiety inventory (form Y).

[46] Huang L, Yang TZ, Ji ZM (2003). Applicability of the Positive and Negative Affect Scale in Chinese. Chin. Mental. Health. J..

[47] Watson D, Clark LA, Tellegen A (1988). Development and validation of brief measures of positive and negative affect: the PANAS scales. J. Pers. Soc. Psychol..

[48] Matsumoto, D. & Ekman, P. *Japanese and caucasian facial expressions of emotion* (*JACFEE*) *and neutral faces* (*JACNeuF*) [CDs] (San Fransisco, CA: Intercultural and Emotion Research Laboratory, Department of Psychology, San Francisco State University, 1988).

[49] al’Absi M (1997). Cardiovascular and neuroendocrine adjustment to public speaking and mental arithmetic stressors. Psychophysiology.

[50] Buchanan TW, Laures-Gore JS, Duff MC (2014). Acute stress reduces speech fluency. Biol. Psychol..

[51] Het S, Rohleder N, Schoofs D, Kirschbaum C, Wolf OT (2009). Neuroendocrine and psychometric evaluation of a placebo version of the ‘Trier Social Stress Test’. Psychoneuroendocrinology.

[52] Zheng XH (1993). State-Trait test report in Changchun. Chin. Mental. Health. J..

[53] Booij SH, Bouma EMC, de Jonge P, Ormel J, Oldehinkel AJ (2013). Chronicity of depressive problems and the cortisol response to psychosocial stress in adolescents: The TRAILS study. Psychoneuroendocrinology.

[54] Semlitsch, H. V., Anderer, P., Schuster, P. & Presslich, O. A Solution for Reliable and Valid Reduction of Ocular Artifacts, Applied to the P300 *Erp. Psychophysiology* **23**, 695–703 (1986). 10.1111/j.1469-8986.1986.tb00696.x 3823345

