# Publisher Correction: Acute Psychological Stress Disrupts Attentional Bias to Threat-Related Stimuli

**DOI:** 10.1038/s41598-017-17923-9

**Published:** 2018-01-05

**Authors:** Caihong Jiang, Tony W. Buchanan, Zhuxi Yao, Kan Zhang, Jianhui Wu, Liang Zhang

**Affiliations:** 1 Key Laboratory of Behavioral Science, Institute of Psychology, Chinese Academy of Sciences, Beijing, China; 2 Department of Industrial Engineering, Tsinghua University, Beijing, 100084 China; 3 Department of Psychology, Saint Louis University, St. Louis, MO USA; 4 College of Psychology and Sociology, Shenzhen University, No.3688, Nanhai Rd., Shenzhen, Guangdong China; 5 Department of Psychology, University of Chinese Academy of Science, Beijing, China

*Scientific Reports* **7**:14607; doi:10.1038/s41598-017-14138-w; Article published online 03 November 2017

The original HTML version of this Article contained a typographical error in the publication date ‘03 November 2017’ which was incorrectly given as ‘06 November 2017’. This has now been corrected in the HTML version of the Article.

