# Publisher Correction: Acute Psychological Stress Disrupts Attentional Bias to Threat-Related Stimuli

**DOI:** 10.1038/s41598-018-22368-9

**Published:** 2018-03-06

**Authors:** Caihong Jiang, Tony W. Buchanan, Zhuxi Yao, Kan Zhang, Jianhui Wu, Liang Zhang

**Affiliations:** 1 Key Laboratory of Behavioral Science, Institute of Psychology, Chinese Academy of Sciences, Beijing, China; 2 Department of Industrial Engineering, Tsinghua University, Beijing, 100084 China; 3 Department of Psychology, Saint Louis University, St. Louis, MO USA; 4 College of Psychology and Sociology, Shenzhen University, No.3688, Nanhai Rd., Shenzhen, Guangdong, China; 5 Department of Psychology, University of Chinese Academy of Science, Beijing, China

Correction to: *Scientific Reports* 10.1038/s41598-017-14138-w, published online 03 November 2017

An error occurred after publication resulting in the inadvertent omission of the Article and Volume numbers from the PDF version of this Article. This error has now been corrected in the PDF version of the Article; the HTML version was correct from the time of publication.

